# Exome and copy number variation analyses of Mayer–Rokitansky–Küster– Hauser syndrome

**DOI:** 10.1038/s41439-018-0028-4

**Published:** 2018-09-27

**Authors:** Kazumi Takahashi, Takahide Hayano, Ryota Sugimoto, Hirofumi Kashiwagi, Mari Shinoda, Yoshihiro Nishijima, Takahiro Suzuki, Shingo Suzuki, Yuko Ohnuki, Akane Kondo, Takashi Shiina, Hirofumi Nakaoka, Ituro Inoue, Shun-ichiro Izumi

**Affiliations:** 1 Department of Obstetrics and Gynecology, Tokai University School of Medicine, Isehara, Kanagawa Japan; 2 Department of Clinical Genetics, Tokai University Hospital, Isehara, Kanagawa Japan; 3 Department of Systems Bioinformatics, Yamaguchi University Graduate School of Medicine, Ube, Japan; 4 Division of Human Genetics, National Institute of Genetics, Mishima, Shizuoka Japan; 5 Department of Molecular Life Science, Division of Basic Medical Science and Molecular Medicine, Tokai University School of Medicine, Isehara, Kanagawa Japan; 6 Perinatal Medical Center, Shikoku Medical Center for Children and Adults, National Hospital Organization, Zentsuji, Kagawa Japan

## Abstract

Mayer–Rokitansky–Küster–Hauser (MRKH) syndrome is characterized by congenital absence of the vagina and uterus. We conducted genome-wide SNP analyses and exome sequencing to detect the causes of MRKH syndrome. We identified de novo variants of *MYCBP2*, *NAV3*, and *PTPN3* in three families and a variant of *MYCBP2* in a sporadic case. Here, we demonstrated the partial genetic makeup of Japanese MRKH syndrome.

Mayer–Rokitansky–Küster–Hauser (MRKH) syndrome is characterized by the congenital absence of the upper two-thirds of the vagina and uterus and occurring at a rate of 1 in 4500 newborn girls. Patients with MRKH syndrome have a normal female karyotype (46XX) and seemingly normal development of secondary sex characteristics and typically present with amenorrhea during adolescence, resulting in problems with sexual intercourse and infertility^1^. This syndrome is classified into type I and type II: type I involves only uterovaginal aplasia, and type II involves uterovaginal aplasia with concomitant defects, such as renal malformations, skeletal malformations, hearing defects, and rare cardiac and digital anomalies^1^. The etiology of this syndrome remains elusive because most of the cases are sporadic with potential underlying heterogenous causes. However, familial aggregation is occasionally observed, and genetic involvement has been reported by several investigators, showing likely autosomal dominant inheritance with incomplete penetrance^1,2^. Frequent genomic rearrangements, such as deletions and duplications, have been identified by array comparative genome hybridization (CGH) analyses; however, a consistent pattern has not been observed, and a responsive gene has not been pinpointed, except for *LHX1* and *HNF1B* on chromosome 17q12^1,3–5^. Because large families that promote linkage and positional cloning are mostly lacking in MRKH syndrome, a candidate gene approach has been utilized for detecting causal genes; thus far, only *WNT4* has been identified in MRKH syndrome with hyperandrogenism^1^.

Recent advances in sequence technologies have propelled genetic analyses of both rare and common diseases at the whole-genome level. We performed genome-wide single-nucleotide polymorphism (SNP) analyses to detect chromosomal rearrangement and exome analysis to identify causal variants in trio-families and sporadic cases.

Informed consent was obtained in accordance with the Declaration of Helsinki, and the study protocol was approved by the Institutional Review Board of Tokai University School of Medicine (12I-03, 17I-32), National Institute of Genetics (nig1608) and Yamaguchi University Graduate School of Medicine (H29-229). Each participant gave written informed consent for the collection of samples and subsequent analyses. A total of 17 specimens (ten patients and seven unaffected individuals from the families) were recruited in this study. Six of the ten patients had type I MRKH syndrome, and four of the ten patients had type II MRKH syndrome (Table S1). We also analyzed samples from healthy parents (mother and father) of two patients (A5 and A7) and samples from healthy family members (mother, father, and sister) of one patient (A6). We designated families of A5, A6, and A7 as Fam01, Fam02, and Fam03, respectively (Figure S1).

Genomic DNA was extracted from the peripheral white blood cells of individuals using the QIAamp DNA Mini Kit (Qiagen) according to the manufacturer’s instructions. Genomic DNA was hybridized using the SureSelect Human All Exon V5 Kit (Agilent) and sequenced using a HiSeq 2500 (Illumina) with 100 or 150 base paired end modules. Sequencing data were mapped to a human genome reference (hg19) using a standard method of BWA, Picard, and GATK, as previously described^6,7^. Variant calling and genotyping were performed using GATK HaplotypeCaller^6^. De novo variants in the three families (Fam01, Fam02, and Fam03) were called using the Trio Calling module of VarScan^8^. Allele frequency and functional information of variants were annotated by ANNOVAR^9^. We also used allele frequencies in the Japanese population for further selection of variants using the Human Genetic Variation Database (HGVD)^10^. After selection of variants, the allele frequencies were manually reviewed in the Integrative Japanese Genome Variation Database (iJGVD)^11^. Single-nucleotide polymorphism (SNP) array experiments using Infinium OmniExpress-24 BeadChips (Illumina) were conducted for nine of the 10 patients with MRKH except for A5. A PennCNV-ParseCNV analysis pipeline^12,13^ was applied to detect structural variations. To identify disease-specific CNV, we used in-house CNV data from individuals without MRKH. For Fam01 (A5, C8, and C9), the array-CGH experiment was conducted using the Agilent Human Genome CGH Microarray 1M Kit.

Three common structural variations were identified by the PennCNV-ParseCNV pipeline (Fig. 1). One amplified structural variation (chr2:10,886,097–10,890,204) was found in A1 and A8. A4 and A6 shared a deleted region of chr8:135,062,170–135,065,947. A7 and A9 shared a deleted region of chr16:5,608,833–5,613,997. Identified structural variations were not large and were not involved in coding regions. No overlapping region in the reported results and *WNT4* region was identified. Because two patients shared the same structural variations at each site, these variations would likely be polymorphic. Therefore, we shifted our focus to single-nucleotide variants (SNVs) and small insertion and deletions (Indels) to evaluate causality using whole-exome analyses mainly in trio-based families.

**Fig. 1:**
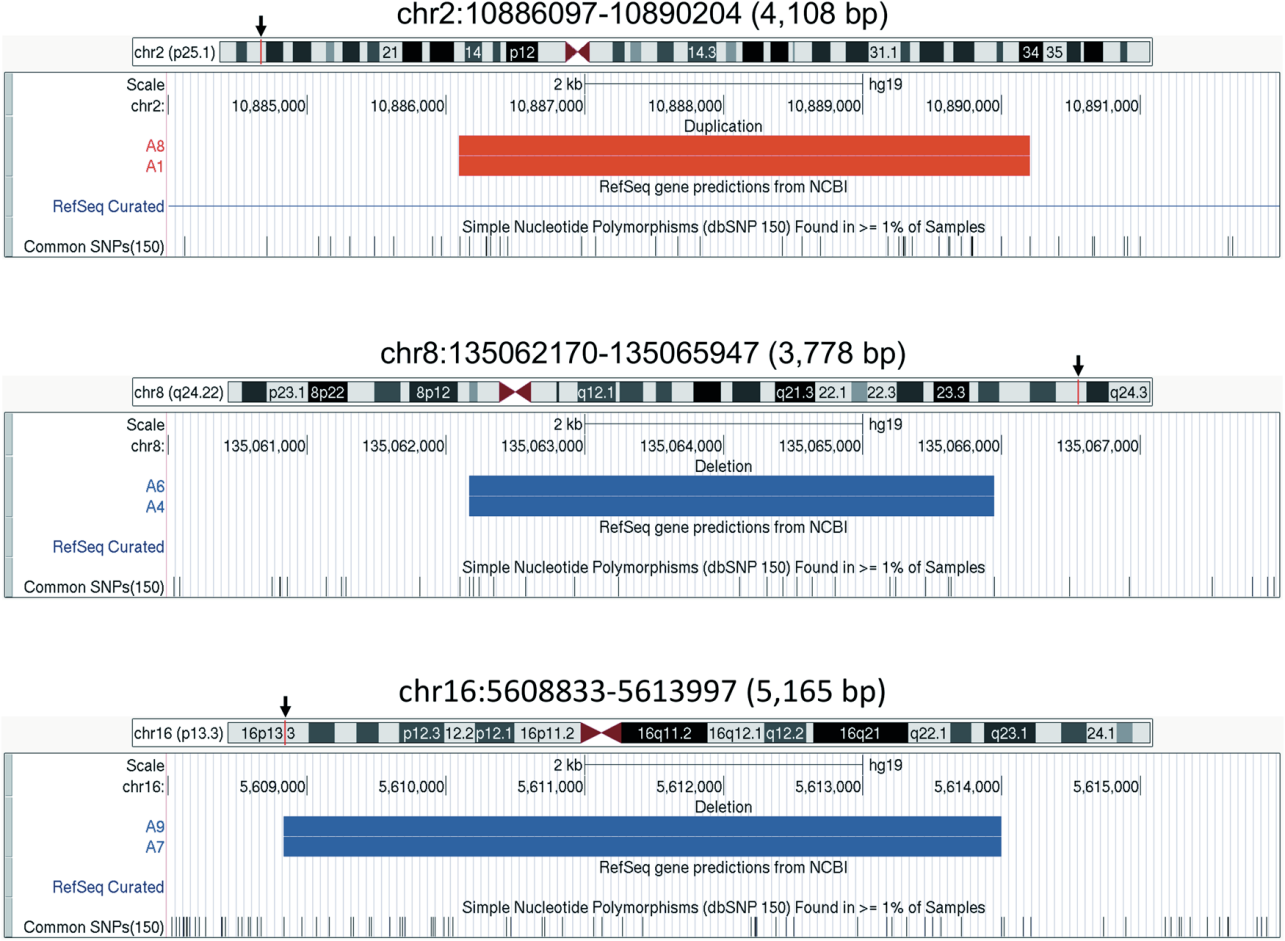
Three common structural variations of MRKH syndrome. (Top) A duplication (4108 bp) in chromosome 2 (chr2p25.1). A1 and A8 had a common duplication in chr2:10,886,097–10,890,204. (Middle) A deletion (3778 bp) in chromosome 8 (chr8q24.22). A4 and A6 had a common deletion in chr8:135,062,170–135,065,947. (Bottom) A deletion (5165 bp) in chromosome 16 (chr16p13.3). A7 and A9 had a common deletion in chr16:5,608,833–5,613,997. Black arrows and red or blue vertical lines indicate the positions of structural variations in chromosome ideograms. All structural variations were visualized using UCSC Genome Browser (hg19)

Ten patients including three patients from trio-based families and seven unaffected individuals from their families were subjected to whole-exome analyses. The mean depth of exome sequencing was ×71 and ranged from ×49 to ×98. Coverage ≥ ×10 was achieved for more than 90% of all the samples (Table S2). A total of 228,135 variants and an average of 95,293 variants (from 91,835 to 97,514) were detected in the 17 samples. After selection of exonic and splicing variants with an allele frequency ≤ 5%, an average of 284 variants (from 247 to 348) remained. Variants were surveyed using the Japanese public database (HGVD), and variants with allelic frequency <0.1% were selected (Table S3 and S4). We could not find any variants in the previously reported MRKH-associated genes (*LHX1*, *HNF1B*, and *WNT4*). To identify de novo variant(s) in the three families, we conducted trio-based de novo variant calling. After manual curation of variants using IGV^14^, we identified three de novo variants in three genes (*MYCBP2*, *NAV3*, and *PTPN3*) (Table S5). We also identified a distinct variant of *MYCBP2* in a sporadic case (A1). Genotypes of all the de novo variants and the sporadic variant of *MYCBP2* were heterozygous, and the positions of the variants are shown in Table 1. Functional predictions of variants in *MYCBP2* (A1 and A5), *NAV3* (A6), and *PTPN3* (A7) are summarized in Table 1. All the identified variants have not been registered in the most updated databases, and all the variants are probably deleterious (especially predicted by MutationTaster and fathmm-MKL). *MYCBP2* is located in chromosome 13q22.3, which has not yet been identified to harbor SNVs and chromosome aberrations in MRKH syndrome. *MYCBP2* encodes an E3 ubiquitin–protein ligase and has not been described in the etiology of uterine and vaginal development^15^. Reduced expression of *MYCBP2* has been observed in acute lymphoblastic leukemia patients^16^, and a deletion mutation in this gene has been associated with developmental abnormality of optical discs, resulting in a rare inherited vision defect^17^. *NAV3* is located on chromosome 12, and it belongs to a neuron navigator family that is expressed in the nervous system^18^. Because *NAV3* variants have not been implicated in diseases and only one patient had the variant, *NAV3* was not considered to be a cause of MRKH. *PTPN3* is located on chromosome 9, and it belongs to a tyrosine phosphatase family^19^. *PTPN3* has multiple functions in cellular process, such as differentiation and growth. Somatic mutation of *PTPN3* can promote cell proliferation and cholangiocarcinoma^20^, but the involvement of this gene in MRKH is not clear.

**Table 1:** Functional predictions of the three de novo variants and MYCBP2 variants in A1

Paitent	A5	A1	A6	A7
Position	chr13:77661732	chr13:77752067	chr12:78400834	chr9:112182816
Gene	*MYCBP2*	*MYCBP2*	*NAV3*	*PTPN3*
NCBI accession	NM_015057	NM_015057	NM_001024383	NM_002829
Protein change	p.T3588A	p.H1719P	p.I506V	p.N401H
Genotype (Het/Homo)	Het	Het	Het	Het
SIFT_score	0.101	0.053	0.068	0.893
SIFT_pred	T	T	T	T
Polyphen2_HDIV_score	0.956	0.980	0.913	0.917
Polyphen2_HDIV_pred	P	D	P	P
Polyphen2_HVAR_score	0.931	0.948	0.891	0.219
Polyphen2_HVAR_pred	D	D	P	B
LRT_score	0.000	0.000	0.001	0.001
LRT_pred	D	D	U	D
MutationTaster_score	1.000	1.000	1.000	0.989
MutationTaster_pred	D	D	D	D
MutationAssessor_score	1.780	0.690	2.565	1.100
MutationAssessor_pred	L	N	M	L
FATHMM_score	1.610	1.710	1.200	1.760
FATHMM_pred	T	T	T	T
PROVEAN_score	−2.410	−3.690	−0.680	0.100
PROVEAN_pred	N	D	N	N
MetaSVM_score	−1.003	−1.036	−0.731	−0.981
MetaSVM_pred	T	T	T	T
MetaLR_score	0.121	0.100	0.218	0.130
MetaLR_pred	T	T	T	T
M-CAP_score	0.010	0.009	0.011	0.013
M-CAP_pred	T	T	T	T
fathmm-MKL_coding_score	0.994	0.987	0.994	0.975
fathmm-MKL_coding_pred	D	D	D	D
GERP + + _RS	5.440	5.720	5.490	4.800

Twelve functional prediction scores (SIFT, Polyphen2_HDIV, Polyphen2_HVAR, LRT, MutationTaster, MutationAssessor, FATHMM, PORVEAN, MetaSVM, MetaLR, M-CAP, and fathmm-MKL) and one evolutionally conservation score (GERPP + + _RS) were annotated using ANNOVAR

*Het* Heterozygote, *Homo* Homozygote, *T* tolerated, *D* damaging or deleterious, *P* probably damaging, *B* benign, *U* unknown, *L/N* non-functional, *M* medium

Genetic approaches to identify the genetic causalities for MRKH syndrome have not been successful. Thus, the focus should be on epigenetic and environmental factors underlying the disease. Indeed, discordant phenotype in twin sisters has been reported, indicating more heterogenous characteristics of the syndrome. Therefore, genetic and nongenetic factors need to be investigated for full understanding of MRKH syndrome.

We identified two mutations in *MYCBP2* in two patients (A1 and A5). In particular, one patient (A5) showed a de novo mutation. The functional involvement of *MYCBP2* in the etiology of MRKH syndrome needs to be further investigated.

## Electronic supplementary material

Table S1

Figure S1

Table S2

Table S3

Table S4

Table S5

## Data Availability

The relevant data from this Data Report are hosted at the Human Genome Variation Database at 10.6084/m9.figshare.hgv.2378 10.6084/m9.figshare.hgv.2381 10.6084/m9.figshare.hgv.2384 10.6084/m9.figshare.hgv.2387
