# Supplementary material for: Exome and copy number variation analyses of Mayer–Rokitansky–Küster– Hauser syndrome
Source: Hum Genome Var. 2018 Sep 27;5:27. doi: 10.1038/s41439-018-0028-4 (PMC6160444; doi:10.1038/s41439-018-0028-4)
Supplement: Supplementary file 1 — Table S1 [file 41439_2018_28_MOESM1_ESM.pdf]

Table S1. Clinical information of 10 patients.

Patient	Family/Sporadic	Phenotype	Genital	Renal	Skeleton	Heart	Hearing
A1	Sporadic	Type I	Rudimentary uterus (bilateral), Vaginal agenesis	Normal	Normal	Normal	Normal
A2	Sporadic	Type II	Rudimentary uterus, Vaginal agenesis	Normal	Scoliosis	Normal	Normal
A3	Sporadic	Type I	Rudimentary uterus (bilateral), Vaginal agenesis	Normal	Normal	Normal	Normal
A4	Sporadic	Type I	Rudimentary uterus (bilateral), Vaginal agenesis	Normal	Normal	Normal	Normal
A5	Trio	Type I	Rudimentary uterus (Rt.), Vaginal agenesis	Normal	Normal	Normal	Normal
A6	quartet	Type I	Rudimentary uterus (bilateral), Vaginal agenesis	Normal	Normal	Normal	Normal
A7	Trio	Type II	Rudimentary uterus (bilateral), Vaginal agenesis	Lt. kidney agenesis	Normal	Normal	Normal
A8	Sporadic	Type II	Rudimentary uterus, Vaginal agenesis	Normal	Scoliosis, Funnel chest	Brady cardia	Rt. hearing impairment
A9	Sporadic	Type I	Rudimentary uterus (bilateral), Vaginal agenesis	Normal	Normal	Normal	Normal
A10	Sporadic	Type II	Rudimentary uterus (bilateral), Vaginal agenesis	Normal	Scoliosis	Normal	Normal

Genital, genital system aplasia; Renal, renal system aplasia; Skeleton, skeletal malformations; Heart, cardiac anomalies; Hearing, hearing defects.
