# Supplementary material for: Exome and copy number variation analyses of Mayer–Rokitansky–Küster– Hauser syndrome
Source: Hum Genome Var. 2018 Sep 27;5:27. doi: 10.1038/s41439-018-0028-4 (PMC6160444; doi:10.1038/s41439-018-0028-4)
Supplement: Supplementary file 2 — Figure S1 [file 41439_2018_28_MOESM2_ESM.pptx]

## Slide 1
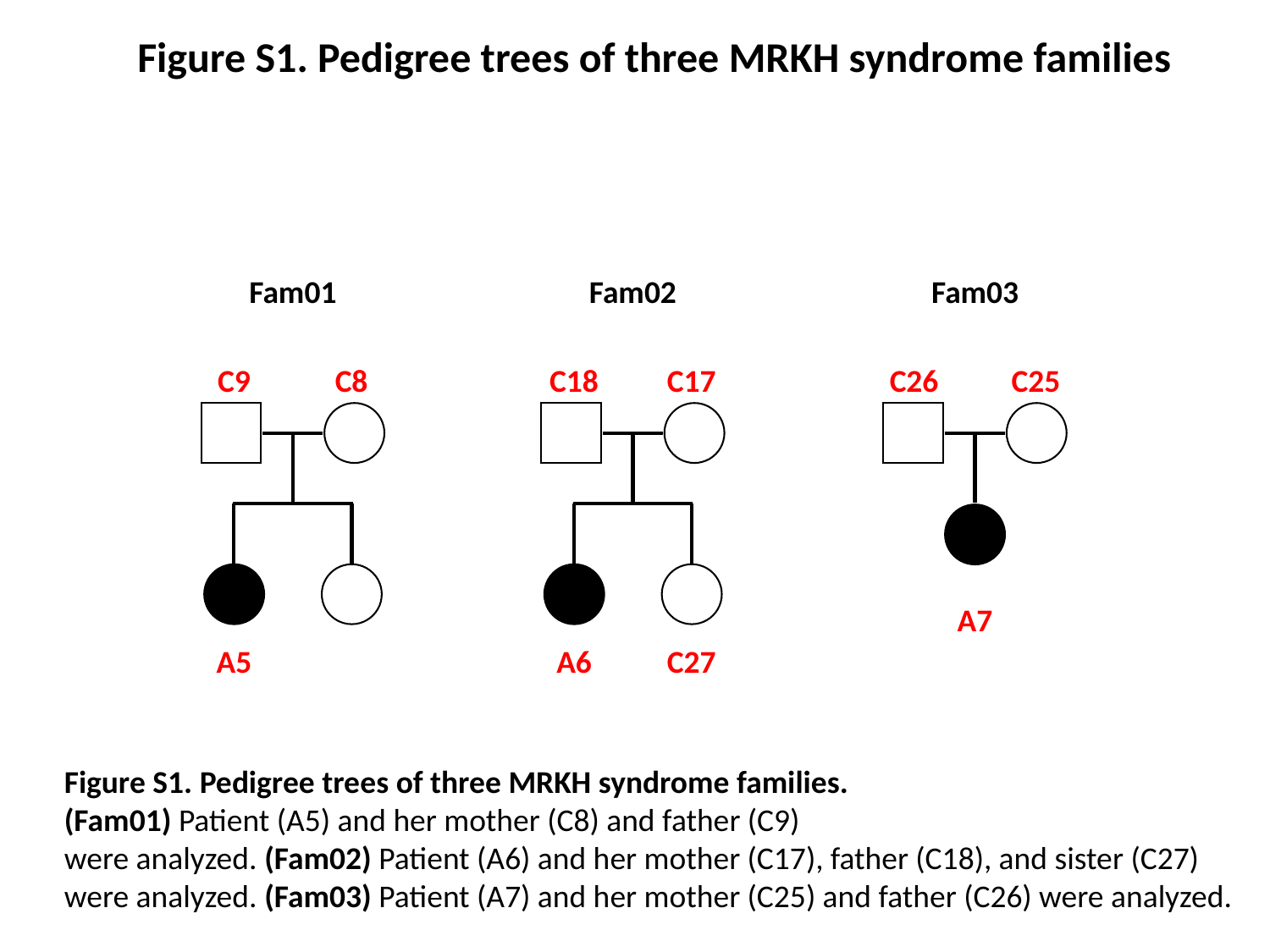

Figure S1. Pedigree trees of three MRKH syndrome families
Fam01
Fam02
Fam03
C9
C8
A5
C18
C17
A6
C27
C26
C25
A7
Figure S1. Pedigree trees of three MRKH syndrome families.
(Fam01) Patient (A5) and her mother (C8) and father (C9)
were analyzed. (Fam02) Patient (A6) and her mother (C17), father (C18), and sister (C27)
were analyzed. (Fam03) Patient (A7) and her mother (C25) and father (C26) were analyzed.
