# Supplementary material for: Exome and copy number variation analyses of Mayer–Rokitansky–Küster– Hauser syndrome
Source: Hum Genome Var. 2018 Sep 27;5:27. doi: 10.1038/s41439-018-0028-4 (PMC6160444; doi:10.1038/s41439-018-0028-4)
Supplement: Supplementary file 6 — Table S5 [file 41439_2018_28_MOESM6_ESM.pdf]

Table S5. De novo variants found in the three MRKH syndrome families.

Patient	Position	Gene	NCBI accession	cDNA change	Protein change	HGDV	iJGVD
A5	chr13:77661732	*MYCBP2*	NM_015057	c.A10762G	p.T3588A	NA	NA
A6	chr12:78400834	*NAV3*	NM_001024383	c.A1516G	p.I506V	NA	NA
A7	chr9:112182816	*PTPN3*	NM_002829	c.A1201C	p.N401H	NA	NA

cDNA changes and protein changes were annotated based on nucleotide database (NCBI accession) and chromosomal position (hg19) using ANNOVAR. All the variants were novel variants in the two public database of Japanese (HGVD and iJGVD). NA, Not applicable.
